# Development of Biolayer Interferometry (BLI)-Based Double-Stranded RNA Detection Method with Application in mRNA-Based Therapeutics and Vaccines

**DOI:** 10.3390/pharmaceutics16091227

**Published:** 2024-09-19

**Authors:** Dharia Sara Silas, Bindiya Juneja, Keerat Kaur, Muralikrishna Narayanareddy Gari, Yingjian You, Youmi Moon, Yizhuo Chen, Srishti Arora, Johanna Hansen, Kathir Muthusamy, Yue Fu, Nisha Palackal, Erica A. Pyles

**Affiliations:** 1 Protein Biochemistry, Regeneron Pharmaceuticals, Tarrytown, NY 10591, USA; 2 Regeneron Genetic Medicines, Regeneron Pharmaceuticals, Tarrytown, NY 10591, USA; 3 Vaccine Technology, Regeneron Pharmaceuticals, Tarrytown, NY 10591, USA

**Keywords:** biolayer interferometry (BLI), double stranded RNA (dsRNA), binding affinity, uridine modification, FHV B2, RNA detection methods

## Abstract

**Background:** In vitro-transcribed (IVT) mRNA has been established as a promising platform for therapeutics and vaccine development. Double-stranded RNA (dsRNA) is a major impurity of IVT mRNA and can trigger unfavored immune responses, potentially causing adverse events in patients. Existing dsRNA detection and quantitation methods, such as gel electrophoresis, ELISA, or homogeneous time-resolved fluorescence (HTRF), have low sensitivity or are time-consuming. A recently published lateral flow immunoassay (LFSA) was shown to be fast, but it lacks the sensitivity for dsRNA with uridine modifications. **Methods:** In this study, we provided a possible explanation for the reduced sensitivity of existing quantitation methods for dsRNA with modified uridines by characterizing the binding affinities of commonly used anti-dsRNA antibodies. Then, a rapid and sensitive biolayer interferometry (BLI) dsRNA detection assay utilizing Flock House Virus (FHV) B2 protein was developed to overcome the challenges in dsRNA detection and the reduced sensitivity. **Results:** This assay allows the detection of dsRNA with different uridine modifications (ψ, m1ψ, 5 moU) with similar sensitivity as dsRNA without modification. Furthermore, we demonstrated this method can be used to quantify both short and long dsRNA, as well as hairpin-structured dsRNA, providing a more comprehensive detection for dsRNA impurities. Moreover, we applied this assay to monitor dsRNA removal through a purification process. **Conclusions:** Taken together, this BLI method could enable real-time monitoring of impurities in IVT mRNA production to prevent immunogenicity stemming from dsRNA.

## 1. Introduction

Despite current challenges such as the need for ultracold storage and tissue-specific delivery, mRNA-based vaccines and therapeutics have showcased the capacity and potential to be mRNA-based platforms for drug discovery and development. There are numerous ongoing clinical studies evaluating mRNA platforms for enzyme replacement, antiviral, and other applications. mRNA generated for these applications is mainly produced through in vitro transcription (IVT) using T7 RNA polymerase [1,2]. Due to the nature of T7 RNA polymerase, transcription abortion or slippage can occur, which can produce either short or long dsRNA byproducts [3,4,5,6,7]. Because the double-stranded structure is a hallmark feature of foreign viral nucleic acid, once they enter human cells, the dsRNA impurities can (1) activate dsRNA-dependent enzymes, such as oligoadenylated synthetase (OAS), RNA-activated protein kinase (PKR), and RNA-specific adenosine deaminase (ADAR), causing inhibition of protein translation [8,9,10]; (2) stimulate endogenous dsRNA sensors, including Toll-like receptor 3 (TLR3), melanoma differentiation-associated protein 5 (MDA5), and retinoic acid-inducible gene I (RIG-I), resulting in the activation of innate immunity and secretion of various cytokines, i.e., interleukin-6 (IL-6), tumor necrosis factor-α, and type I interferons [11,12]. Therefore, dsRNA impurities have the potential to affect the potency and safety of mRNA drug products. To reduce the immunogenicity effect of dsRNA, three approaches are commonly used: (1) replace uridines in mRNA with modified uridines, such as m1ψ or 5 [13], (2) optimize the IVT reaction condition or T7 polymerase to reduce dsRNA formation [14,15,16], or (3) subject IVT mRNA to further purification [17,18,19]. To ensure the success of these approaches, it is critical to implement a rapid and robust detection and quantitation method to monitor the level of dsRNA.

There are several commonly used dsRNA detection and quantitation methods, including Dot blot, ELISA (J2 or K1 antibody) [20], homogeneous time-resolved fluorescence (HTRF) [21], and Bioluminescence assay (Promega). There is also a recent report using lateral flow immunoassay (LFSA) for dsRNA quantitation [22]. Additional biophysical techniques could also be explored for dsRNA detection, including surface plasma resonance (SPR) or atomic force microscopy (AFM), given their success in measuring RNA interaction [23,24]. Except for the Promega Lumit^®^ assay, which uses the dsRNA binding domain (dsRBD) from Protein kinase R (PKR), all other assays utilize one antibody, or a combination of antibodies raised against dsRNA or poly(I:C) [20,25], including but not limited to J2, K1, or J5. Although these antibodies have been used extensively for more than 30 years, little is known about their binding affinity to dsRNA-containing modified uridines. Furthermore, it was indicated in the literature that the K1 antibody has a weaker binding affinity to dsRNA [20]. Indeed, a combination of K1 and J5 antibodies used in LFSA has reduced detection sensitivity for m1ψ-modified dsRNA [22]. Taken together, these results raise the question of whether existing ELISA assays, or more generally speaking, assays using existing antibodies, can reliably report the dsRNA levels in mRNA with m1ψ modification, which is the most utilized modification in mRNA-based therapeutics or vaccines.

Flock House Virus (FHV) is an insect virus that contains a positive sense RNA genome. For insect hosts, the RNA silencing pathway was utilized as a self-defense mechanism against viral infection. The RNA silencing pathway depends on the formation of an RNA-inducing silencing complex (RISC) which targets siRNA derived from the FHV genome to specifically degrade the viral genome [26]. FHV encodes a protein called B2, which acts as a countermeasure against RNA silencing [27]. The suppression mechanism of RNA silencing by B2 has been shown to be dual-mode: (1) by binding to the double-strand RNA formed during viral genome replication, which can block DICER cleavage, and (2) by binding to siRNA from the DICER cleavage, sequestering its incorporation into the RISC [27]. It has been shown that the B2 protein is a potent dsRNA binder that can bind to a dsRNA as short as 17 bp and has a binding mode different from canonical dsRBD [27,28]. It has been proposed that multiple B2 proteins can bind to one dsRNA at the same time, with stoichiometry increasing according to the length of the dsRNA [27]. Since its discovery, the B2 protein has been utilized to study dsRNA in living cells, including but not limited to dsRNA location and distribution, due to its length and sequence-independent binding to dsRNA [29,30]. For this study, we intend to expand the application of the FHV B2 protein to dsRNA quantitation.

Biolayer interferometry (BLI) is a well-established platform known for its rapid and robust titer/concentration measurement and quantification of various biologics, including but not limited to antibodies, adeno association viruses (AAV), and host cell proteins (HCPs). BLI has various practical advantages, including rapid read-out, simple operation, and low requirements for the sample process. Additionally, BLI’s versatility extends to measuring the binding affinity for various RNA–protein interactions. In this study, we first characterized the binding affinity of FHV B2 to dsRNA (without or with modified nucleosides), as well as the binding affinity of J2 or K1 antibodies to FHV B2. We then leveraged the specificity of B2 to dsRNA to develop a novel BLI dsRNA detection method. Altogether, our BLI method allows rapid and quantitative monitoring of dsRNA, regardless of nucleoside modification, in IVT mRNA products and can be easily implemented as a release method for mRNA drug product manufacturing.

## 2. Material and Methods

### 2.1. Reagents

Nuclease-free water (catalog number AM9932), 1 M Tris, pH 8.0 (catalog number AM9855G), 5 M NaCl (catalog number AM9759), 0.5 M EDTA, pH 8.0 (catalog number AM9260G), and yeast tRNA (catalog number AM7119) were all RNase-free grade from Thermofisher Scientific (Carlsbad, CA, USA). Tween^TM^ 20 (catalog number 85113) was also purchased from Thermofisher Scientific (Rockford, IL, USA).

### 2.2. Recombinant B2 Protein Construct Design, Expression, and Purification

Residues 1–73 from FHV B2 protein (Uniprot P68831) were cloned into pET30a vector. Two different constructs of B2 were made: one with N-terminal 6X His tag (refer as B2) and the second one with N-terminal 6X His tag and C-terminal GS linker followed by an AviTag (refer as B2-Avi). Protein was expressed in *E.coli* BL21(DE3) and purified using a two-step purification process (Ni column + Superdex 75 column). For B2-Avi, BirA plasmid was co-transfected to enable co-translational biotinylation of B2-Avi protein (referred to as B2-Biotin). The identity and purity of each purified protein were confirmed by SDS-PAGE and LC-MS. All vector design, construct, protein expression, and purification were performed by Genscript (Genscript USA, Piscataway, NJ, USA).

### 2.3. Preparation of dsRNA Standards

A list of dsRNA standards, prepared by CATUG Biotechnology (Suzhou, China), is provided in Supplementary Table S1. The standards vary in length and include uridine modifications. These dsRNA standards were prepared by annealing complementary ssRNA strands produced from IVT. A hairpin dsRNA which contains a 100A poly(A) tail and 70 bp complementary sequence before and after the poly(A) tail was also included in this study, which was designed to better mimic the dsRNA byproduct from IVT. Prior to use, the purity of each dsRNA standard was confirmed by capillary electrophoresis (CE) (Supplementary Table S2). The double-strand content for each dsRNA standard was confirmed by HTRF assay and ELISA.

### 2.4. IVT mRNA Preparation

PCR products generated with plasmid templates (Genscript USA, Piscataway, NJ, USA) or linearized plasmids were used as the template for mRNA generation. mRNAs were generated by in vitro transcription with a customized ribonucleoside blend of guanosine triphosphate, adenosine triphosphate, cytidine triphosphate (Thermofisher scientific, Carlsbad, CA, USA), and N1-methyl-pseudouridine-5′-triphosphate (m1ψ) (TriLink Biotechnologies, San Diego, CA, USA) and T7 RNA polymerase (Thermofisher scientific, Carlsbad, CA, USA); 5′ cap was added during the in vitro transcription reaction using the Cleancap AG (3′Ome) reagent (Trilink Biotechnologies, San Diego, CA, USA). The mRNA was either purified with tangential flow filtration or silica beads. The concentration of the purified mRNA was determined either with the NanoDrop spectrometer or Qubit (both from Thermofisher scientific, Carlsbad, CA, USA).

### 2.5. ssRNA and dsDNA Preparation

ssRNA (100-nt) was chemically synthesized by Integrated DNA Technologies (IDT, Coralville, IA, USA). dsDNA was prepared from a plasmid template using PCR. Briefly, PCRs were performed by KAPA HiFi HotStart ReadyMix (Roche, Indianapolis, IN, USA). After the linear dsDNA was generated, it was purified by QIAquick PCR purification kit (QIAGEN, Germantown, MD, USA), and the purity was confirmed by the 1% Invitrogen E-Gel Agarose Gel through E-Gel™ Power Snap Electrophoresis System (Thermo Fisher Scientific, Waltham, MA, USA).

### 2.6. CE Analysis of dsRNA Purity

PA800 Plus system (SCIEX, Framingham, MA, USA) was used for CE analysis. The dsRNA ladder sample was prepared by adding 99 µL of nuclease-free water to 1 µL dsRNA Ladder (NEB, Ipswich, MA, USA). The samples were diluted to 5 ng/mL in nuclease-free water. An aliquot of 90 μL of each prepared sample was transferred to a sample vial for injection. A bare fused-silica capillary (30.2 cm) with an inner diameter of 50 μm was used. The separation buffer was composed of 1.5% polyvinylpyrrolidone (Millipore Sigma, St. Louis, MO, USA) in 1X TBE (Millipore Sigma, St. Louis, MO, USA). The detection window was made at 10.2 cm from the outlet of the capillary to make an effective separation length of 20 cm. The sample chamber temperature was set to 10 °C, with the capillary temperature at 30 °C. The sample was injected under a voltage of 3 kV for 3 s with reversed polarity. The electrophoretic separation was carried out with a voltage of 6 kV with reversed polarity for 22 min. Laser-induced fluorescence detection was used to monitor the separation with the excitation wavelength of 488 nm and emission wavelength of 520 nm.

### 2.7. HTRF Assay

HTRF was performed using a viral dsRNA detection kit (Revvity, Waltham, MA, USA, catalog number 64RNAPEG) and following the manufacturer’s protocol. Briefly, the provided standard (5 µg/mL) was prepared in triplicate at 100 ng/mL and serially diluted for 7 concentrations and a blank. The samples (dsRNA or mRNA) were diluted within the linear range of the standard curve. An aliquot of 10 µL of each sample and standard was transferred to a small volume detection white microplate. The detection antibodies (Eu Cryptate and d2) were diluted 50-fold in 1X detection buffer. Just before addition, the detection antibodies were mixed 1:1. This mixture was then added to the plate at 10 µL per well. The plate was then sealed and incubated overnight at 4 °C and read the following day at 665 nm and 620 nm (excitation at 337 nm). A ratio was calculated from the acceptor and donor emission signals to produce a standard curve and quantify the dsRNA level in the samples.

### 2.8. J2 ELISA

J2 ELISA was performed using an ELISA kit (Exalpha, Shirley, MA, USA, catalog number 10613005). The ELISA plate was coated with J2 antibody diluted in PBS and incubated at 4 °C overnight. After coating, the plate was blocked by blocking buffer (1% BSA in PBS + 0.2% NaN_3_) for two hours. Poly (I:C) dsRNA positive control (30 ng dsRNA/well starting and serially diluted 1:3 for 11 concentrations and one blank) and diluted dsRNA or mRNA sample (prepared in STE Buffer: 0.1 M NaCl, 1 mM EDTA, 50 mM Tris-HCl, pH 7.0) were added to the plate (100 µL per well) after plate wash. The plate was incubated for one hour. Undiluted K1 antibody was added to the plate, and then the plate was incubated for one hour before the secondary antibody (HRP-conjugated goat antimouse F(ab)2 fragment) was diluted (1:16,150) and added to the plate (100 µL per well). After one hour of incubation, TMB substrate was added to the plate and incubated for 5–60 min in the dark. The reaction was monitored using a BioTek Synergy Neo2 Reader. When the 650 nm absorbance reached the optimum level, the reaction was stopped, and the absorbance was measured at 450 nm.

### 2.9. BLI Method for Binding Affinity Measurement

Octet^®^ RED384 (Sartorius, Fremont, CA, USA) was used for binding affinity measurement. Briefly, streptavidin biosensors (SA biosensors, Sartorius, Fremont, CA, USA) were first hydrolyzed and equilibrated with assay buffer (10 mM Tris, pH 8.0, 100 mM NaCl, 0.1 mg/mL tRNA, 0.1 mM EDTA, 0.05% Tween-20) for at least 20 min before being used for each experiment. B2-Biotin was diluted in assay buffer to 5 µg/mL and was then captured on SA biosensor to a level > 4 nm to create a dense surface of B2 to enable the avidity binding to dsRNA. This B2 surface was then used to capture dsRNA to a level of ~ 0.3 nm. Binding responses were generated by serial titration of B2 (1017 nM to 16 nM for dsRNA without modification and 2543 nM to 40 nM for all modified dsRNA) to the dsRNA surface with a blank control. For the B2 protein, steady-state analysis (Octet^®^ BLI Analysis v12.2 and Graphpad Prism) was used for the binding affinity determination. The model used for binding affinity (K_D_) determination was as follows:

(1) Response=Rmax×[B2 concentration](KD+B2 concentration)BLI dsRNA detection assay

Octet^®^ RED384 (Sartorius) was used for BLI dsRNA detection development. Briefly, SA biosensors were first hydrolyzed and equilibrated with assay buffer (10 mM Tris, pH 8.0, 100 mM NaCl, 0.1 mg/mL tRNA, 0.1 mM EDTA, 0.05% Tween-20) for at least 20 min before being used for each experiment. B2-Biotin protein was first diluted to 5 µg/mL in assay buffer (10 mM Tris, pH 8.0, 100 mM NaCl, 0.1 mg/mL tRNA, 0.1 mM EDTA, 0.05% Tween-20) and was captured on SA biosensors to a level > 4 nm to create a dense surface for avidity binding to dsRNA. The dsRNA standard curve was generated by serial titration of dsRNA with a concentration range of 4 µg/mL–0.24 ng/mL and a blank control. The dsRNA level in IVT mRNA was determined from the binding response from serially diluted mRNA samples (40 to 5 µg/mL of mRNA) to the B2 surface.

### 2.10. Specificity and Interference Testing of BLI dsRNA Detection Assay

For specificity testing, 20 µg/mL of ssRNA and dsDNA were tested using BLI detection assay, together with 0.2 µg/mL of 700 bp dsRNA (U or m1ψ), 142 bp dsRNA (Jena Biosciences, Jena, Germany), and Poly(I:C) as positive control (Sigma-Aldrich, St. Louis, MO, USA). Assay steps were as described above for dsRNA detection assay. For interference testing, 0.2 ug/mL of 700 bp dsRNA (U or m1ψ) was mixed with 100-fold or 200-fold (*w*/*w*) of ssRNA or dsDNA. The mixture was tested using dsRNA detection assay as described above.

### 2.11. Ion Pair Reverse-Phase FPLC Purification

Reverse-phase purification for IVT mRNA samples was performed using a AKTA Pure FPLC (Cytiva, Uppsala, Sweden) coupled with an RNAsep semipreparative column (21.2 mm × 100 mm, ADS Biotec, Omaha, NE, USA, catalog number RPC-99-2110) with mobile phase A (MPA, ADS Biotec, catalog number 553421): 0.1 M triethylamine acetate (TEAA) pH 7.0 and mobile phase B (MPB, ADS Biotec, catalog number 553422): 0.1 M TEAA, 25% acetonitrile, pH 7.0. The column was equilibrated with 62% MPA and 38% MPB. The gradient used for purification started at 38% MPB with a linear gradient to 60% MPB in 6-column volume (CV) with UV_260_ and UV_280_ monitoring. Fractions containing purified mRNA were buffer exchanged to nuclear-free water to remove organic solvents.

## 3. Results

### 3.1. Recombinant B2 Protein Binds to Long dsRNA with High Affinity

The binding affinity of B2 binding to short dsRNA has been estimated using electrophoretic mobility shift assay (EMSA) [27]. It was previously demonstrated that dsRNA between 17 and 25 bp in length exhibited similar binding affinity to B2 [27]. Although multiple studies have utilized B2 for the in vitro or in vivo study of dsRNA [29,30], there is limited information on the affinity of B2 binding to longer dsRNA, possibly due to the aggregation of the complex formed between B2 and longer dsRNA when they interact. Both short and long dsRNA impurities have been found as a byproduct of mRNA made from the IVT process [3,14]. Thus, a BLI method was developed to assess the binding affinities of B2 towards both short and long dsRNA (Supplementary Figure S1). As illustrated in Supplementary Figure S1, once captured by B2, the immobilized dsRNA surface is very stable, thus providing a stable baseline for affinity measurement. By immobilizing dsRNA on the biosensor, we can treat each binding site on the dsRNA as independent, which would enable a 1:1 binding model for binding affinity measurement.

**Figure 1:**
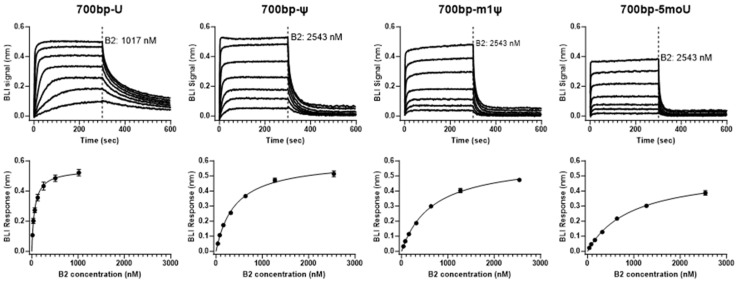
Determination of B2 binding affinity for dsRNA with different modifications. Binding sensorgrams and binding affinity determination of B2 for 700 bp dsRNA with different uridine modifications. (**Top**) (from left to right): representative binding sensorgrams of B2 with 700 bp-U, 700 bp-ψ, 700 bp-m1ψ or 700 bp-5 moU dsRNA. (**Bottom**): corresponding steady-state analysis using binding sensorgrams on the top (BLI signal plotted against B2 concentration) was utilized to determine B2 to dsRNA binding affinity (K_D_). All experiments were performed in triplicate.

Using this method, we measured the binding affinity of B2 for 700 bp-U dsRNA (Figure 1 and Table 1). Due to the fast on-rate of the binding kinetics, steady-state analysis was used to determine the K_D_ value for this interaction, i.e., binding responses (290 to 295 s) were averaged and plotted against B2 concentration. The determined K_D_ value for B2 towards 700 bp-U was ~60 nM. This high-affinity binding further supported using B2 as a dsRNA detection reagent, as shown in a previous report where B2 was used to detect long dsRNA in vitro and in vivo, although, in that study, binding affinity was not determined [29].

### 3.2. B2 Protein and Anti-dsRNA Antibody Binding Affinity towards Long dsRNA Can Be Impacted by Uridine Modifications

To test whether the chemical modification of uridine in dsRNA has any impact on the B2–dsRNA interaction, a set of 700 bp dsRNAs with different uridine modifications (ψ, m1ψ, and 5 moU) were included in the binding affinity measurements. When comparing the binding response from the measurement, it was evident that the interaction between 700 bp-U and B2 was the strongest, followed by 700 bp-ψ and 700 bp-m1ψ, with 700 bp-5 moU being the weakest (Figure 1). Overall, there was a more than 15-fold difference in K_D_ value between 700 bp-U and 700 bp-5 moU, as summarized in Table 1.

The same BLI method was also adapted to measure the binding affinity of two commonly used anti-dsRNA antibodies (J2 and K1) to dsRNA without or with modified uridines. From the determined K_D_ values, J2 exhibited a much stronger binding affinity to dsRNA compared with K1 (Supplementary Table S3A,B). Like B2, uridine modification was also found to have an impact on the J2 (or K1)–dsRNA interaction. A four-fold weaker binding affinity was observed for J2 (or K1) binding to 700 bp-5 moU when compared with binding to 700 bp-U (Supplementary Table S3A,B).

### 3.3. Validation of BLI dsRNA Detection Assay

**Figure 2:**
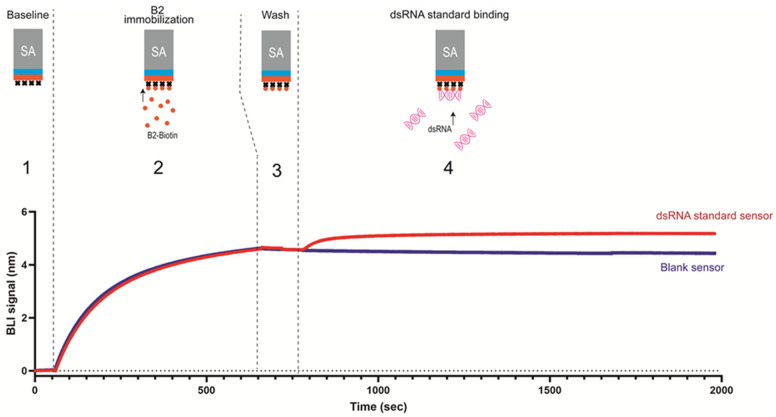
Illustration of BLI dsRNA detection assay. BLI dsRNA detection assay with representative sensorgrams with dotted line separating each step. Step (1) establish baseline in assay buffer; (2) immobilize B2-Biotin on SA sensors; (3) wash to remove unbound B2-Biotin; (4) serially diluted dsRNA binding to B2 surface.

As illustrated in Figure 2, the BLI dsRNA detection assay has only two major steps: (1) B2-biotin was immobilized on SA biosensors to create a dense surface of B2 protein to enable avidity of binding; (2) the B2-immobilized biosensors were then dipped into a sample to detect the presence of dsRNA. The level of dsRNA could be reflected by the binding response at the end of binding sensorgrams. Notably, the total run time is less than 35 min for this assay (per 16 biosensors).

**Figure 3:**
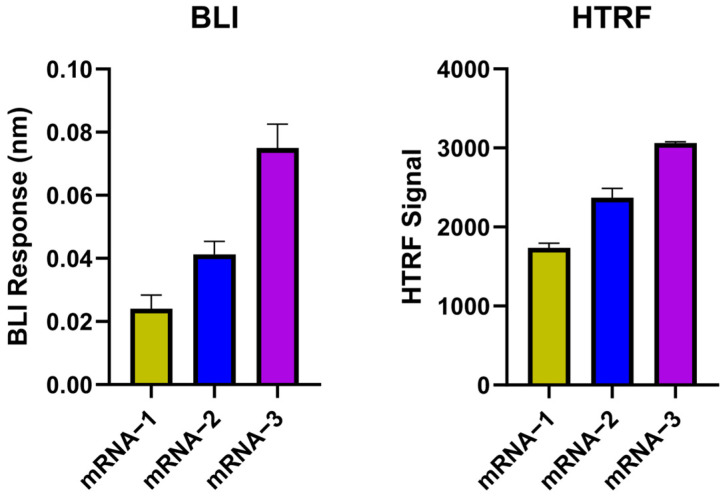
BLI dsRNA detection assay validation with HTRF assay. dsRNA detection by BLI dsRNA detection assay showed a similar trend as HTRF dsRNA assay for three IVT mRNA samples: (**Left**), BLI assay responses for mRNA-1 to mRNA-3 (with blank signal subtracted). (**Right**), HTRF signal (ratio) measured for mRNA-1 to mRNA-3 (with blank signal subtracted). All IVT mRNA samples were tested at 5 µg/mL in triplicates (BLI) and duplicates (HTRF).

To validate the feasibility of the BLI dsRNA detection assay, the BLI responses of three different IVT mRNA transcripts (mRNA-1 to mRNA-3) were compared with the signals from the HTRF assay (Figure 3). Based on BLI responses, mRNA-3 has the highest level of dsRNA, followed by mRNA-2 and then mRNA-1 (Figure 3). These results were consistent with the results from the HTRF analysis of these three mRNA samples, providing confirmation that BLI dsRNA detection assay can reliably detect dsRNA levels in IVT mRNA samples.

### 3.4. Evaluating Specificity and Interference of BLI Method for dsRNA Detection

Besides sensitivity, high specificity is also essential for a successful dsRNA detection method. One major reason is that the IVT mRNA process contains dsDNA (linearized template) and ssRNA. These nucleic acids have a similar chemical composition as dsRNA and are also likely in excess compared with the dsRNA present in IVT mRNA samples.

**Figure 4:**
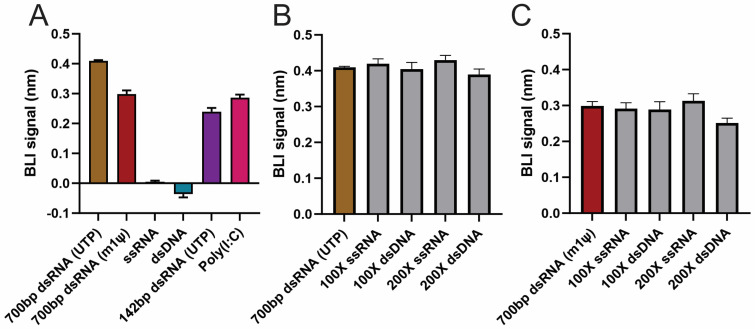
BLI dsRNA detection assay specificity and interference testing. Testing specificity and interference using chemically similar nucleic acids on BLI dsRNA detection assay: (**A**) Specificity testing: BLI signal is shown for 700 bp-U, 700 bp-m1ψ, ssRNA (100-fold), dsDNA (100-fold), 142 bp dsRNA, and Poly(I:C). (**B**) Interference testing: BLI signal of 700 bp-U compared against 700 bp-U in the presence of 100-fold or 200-fold excess of ssRNA or dsDNA. (**C**) Interference testing: BLI signal of 700 bp-U was compared against 700 bp-m1ψ in the presence of 100-fold or 200-fold excess of ssRNA or dsDNA. All experiments were performed in triplicate.

The ability of the BLI dsRNA detection assay to distinguish dsRNA from dsDNA and ssRNA was first evaluated. dsRNA samples, including 700 bp dsRNA (U or m1ψ), 142 bp dsRNA, and Poly(I:C) (positive control), were tested in parallel with dsDNA and ssRNA at 100-fold concentration compared with dsRNA. Minimal or even negative BLI responses were observed for ssRNA and dsDNA samples, even at 100-fold concentration over dsRNA (20 µg/mL vs. 0.2 µg/mL) (Figure 4A). Meanwhile, all dsRNA samples and positive control sample showed decent signal (Figure 4A). These data suggested that this BLI detection method has little cross-reactivity with ssRNA or dsDNA.

The impact of an excessive amount of ssRNA or dsDNA in the dsRNA sample on the signal of the BLI method was then assessed. Two dsRNA standards, 700 bp-U or 700 bp-m1ψ (concentration of 0.2 µg/mL), were tested separately using BLI detection assay in the presence of an increasing ratio of ssRNA or dsDNA (Figure 4B,C). There was no statistically significant difference in BLI responses between samples without or in the presence of ssRNA or dsDNA (Supplementary Table S4), up to 200-fold excess, except for 700 bp-m1ψ with dsDNA, indicating an excessive amount of ssRNA or dsDNA in the dsRNA sample results in minimal interference in the BLI dsRNA detection assay.

### 3.5. Quantitative Detection of the BLI dsRNA Assay

**Figure 5:**
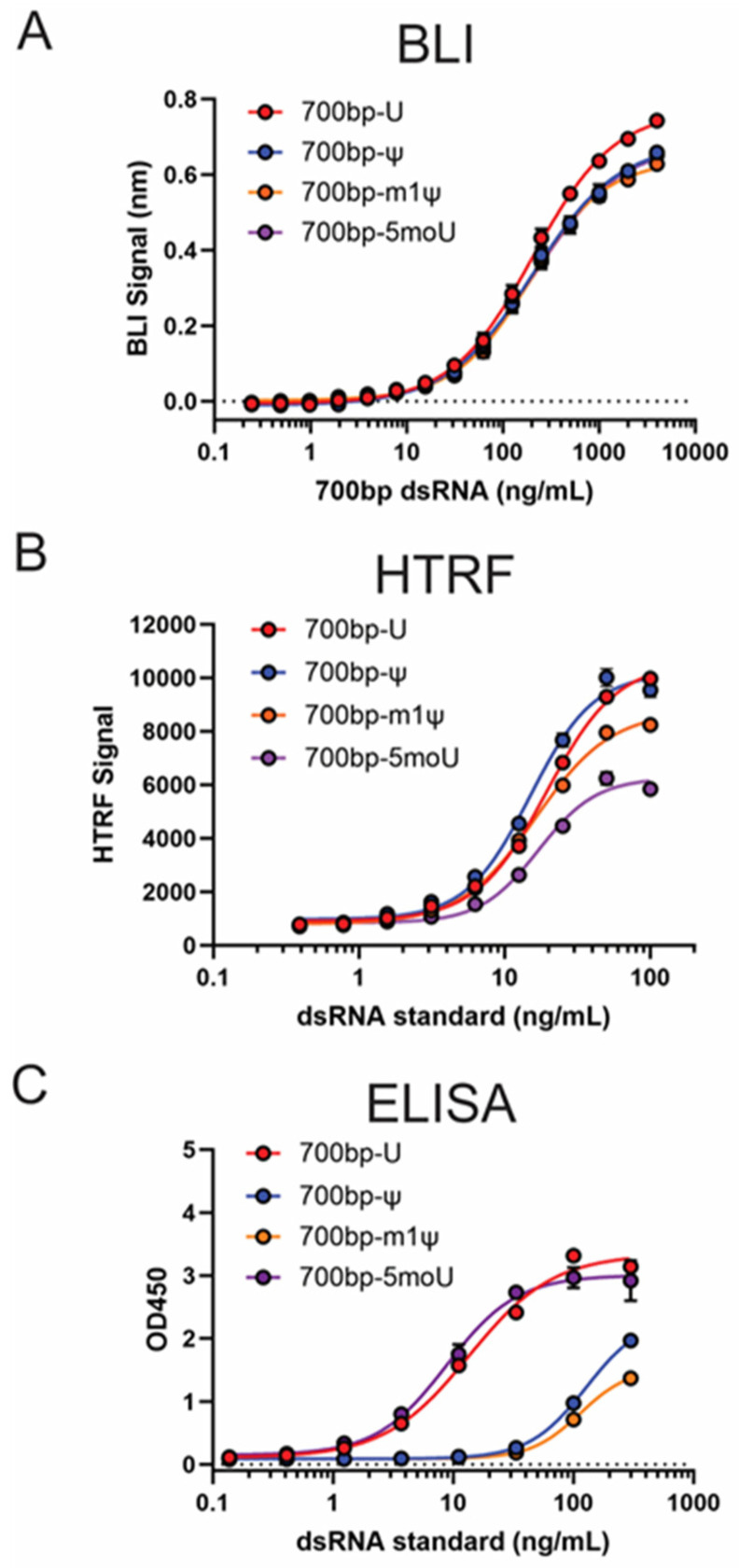
Comparison of impact of uridine modifications on standard curves for BLI, HTRF, and ELISA method for dsRNA quantitation. Comparison of impact of uridine modifications on standard curves for BLI, HTRF, and ELISA methods. From top to bottom, overlay of 700 bp dsRNA standards with different modifications for (**A**) BLI dsRNA detection assay; (**B**) dsRNA quantitation HTRF assay; (**C**) dsRNA quantitation J2 ELISA.

To enable quantitative analysis of dsRNA using the BLI dsRNA detection method, a standard curve needs to be generated using dsRNA standards with known concentration. For 700 bp-U, the standard curve adopted an S-shape response (Supplementary Figure S2), similar to the standard curves from the HTRF assay or ELISA. To better understand whether the utilization of the avidity effect (dense immobilization of B2 on biosensors) helped to achieve the goal of having similar detection sensitivity for dsRNA with or without uridine modifications, standard curves generated from the same concentrations of 700 bp-ψ, 700 bp-m1ψ, and 700 bp-5 moU were compared with the standard curve of 700 bp-U (Figure 5A). Standard curves from modified dsRNA exhibited slightly lower high asymptote but highly similar EC50 values when compared with 700 bp-U (Supplementary Table S5). For the limit of detection (LOD) and limit of quantitation (LOQ), the determined values from these standard curves were highly similar (Supplementary Table S6). These data demonstrated that the detection sensitivity for unmodified and modified dsRNA standards are highly similar using BLI dsRNA detection assay. As a comparison, the set of 700 bp dsRNA standards (U, ψ, m1ψ, and 5 moU) was tested on HTRF assay, as well as J2 ELISA (Figure 5B,C). For HTRF, when compared with 700 bp-U, the standard curves of 700 bp-m1ψ and 700 bp-5 moU showed roughly 20% and 40% lower higher asymptote, respectively (Figure 5B). For J2 ELISA, m1ψ- and ψ-modified dsRNA standard curves showed considerable right-shift (Figure 5C) when compared with nonmodified dsRNA standards.

To test whether the presence of an excessive amount of ssRNA would have an impact on sensitivity, a standard curve for 700 bp-m1ψ dsRNA in the presence of 2.5-fold of ssRNA was generated (Supplementary Figure S3A). No major difference was observed when comparing the standard curves in the presence and absence of ssRNA (Supplementary Figure S3B), suggesting the presence of excess ssRNA in the sample had minimal impact on the quantitation of dsRNA using BLI dsRNA detection assay.

**Figure 6:**
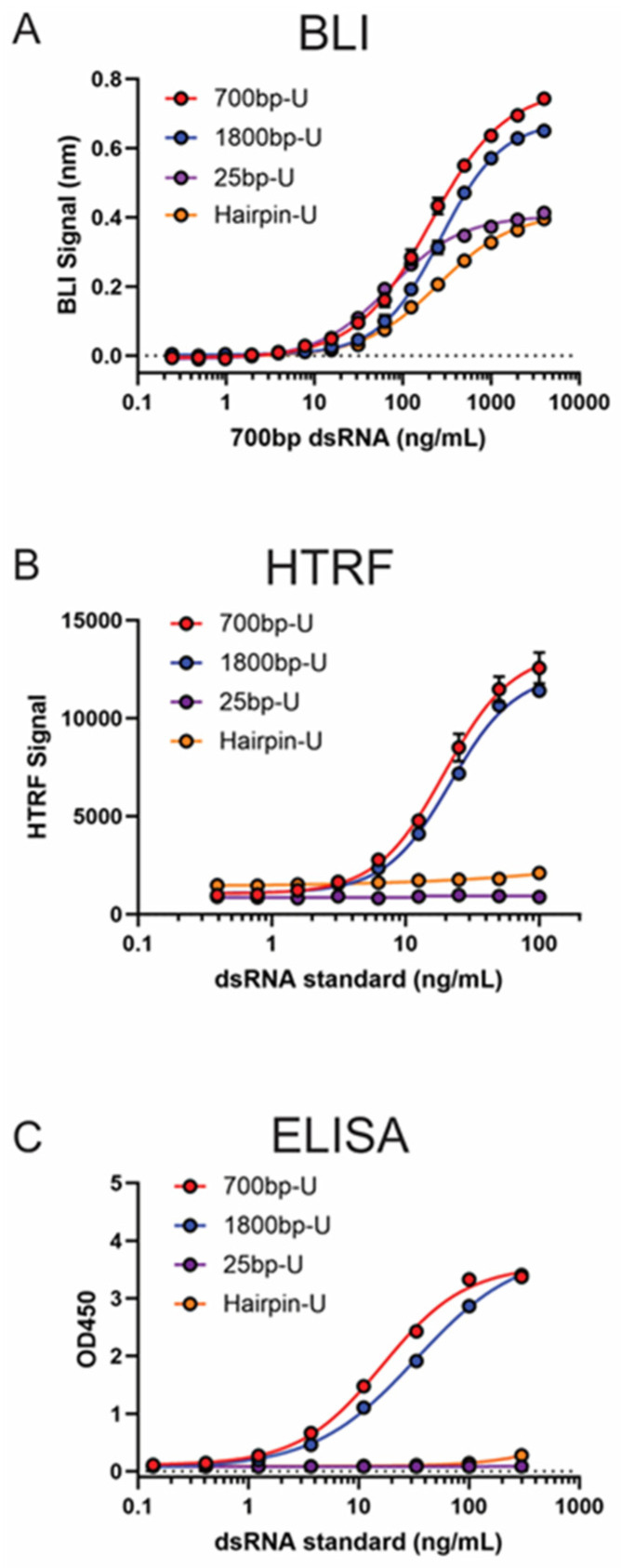
Comparison of dsRNA standards of different lengths on BLI, HTRF, and ELISA methods. Comparison of dsRNA standards of different lengths using BLI, HTRF, and ELISA methods. From top to bottom, overlay of dsRNA standards of different lengths for (**A**) BLI dsRNA detection assay; (**B**) dsRNA quantitation HTRF assay; (**C**) dsRNA quantitation J2 ELISA.

To test whether the BLI dsRNA detection assay was suitable to quantify dsRNA impurities of different lengths and structure features, standard curves for 25 bp dsRNA (U or m1ψ), 1800 bp dsRNA (U or m1ψ), and hairpin dsRNA (U or m1ψ) were generated (Figure 6A and Supplementary Figure S4). Based on a previous study, the length of 25 bp dsRNA is close to the shortest dsRNA that B2 can bind (17 bp) [27]. Although showing a reduction in higher asymptote when compared with longer dsRNA, the BLI dsRNA detection method exhibited comparable sensitivity towards such short dsRNA based on determined LOD and LOQ values (Supplementary Table S6). In contrast to 700 bp or 1800 bp dsRNA standards, which are mostly duplex structures, hairpin dsRNA consists of a normal mRNA followed by a complementary region before and after the poly(A) region. The BLI dsRNA detection assay was able to reasonably quantify the levels of hairpin dsRNA with comparable sensitivity as duplex dsRNA (Supplementary Table S6), as demonstrated by the standard curves generated using the hairpin dsRNA (Supplementary Figure S4). As a comparison, 25 bp-U, 1800 bp-U, and hairpin-U dsRNA standards were also tested using HTRF and J2 ELISA (Figure 6B,C). The results clearly demonstrate that both assays failed to detect 25 bp or hairpin dsRNA.

### 3.6. BLI dsRNA Detection Assay Can Be Used to Monitor Clearance of dsRNA Impurity through Purification Process

**Figure 7:**
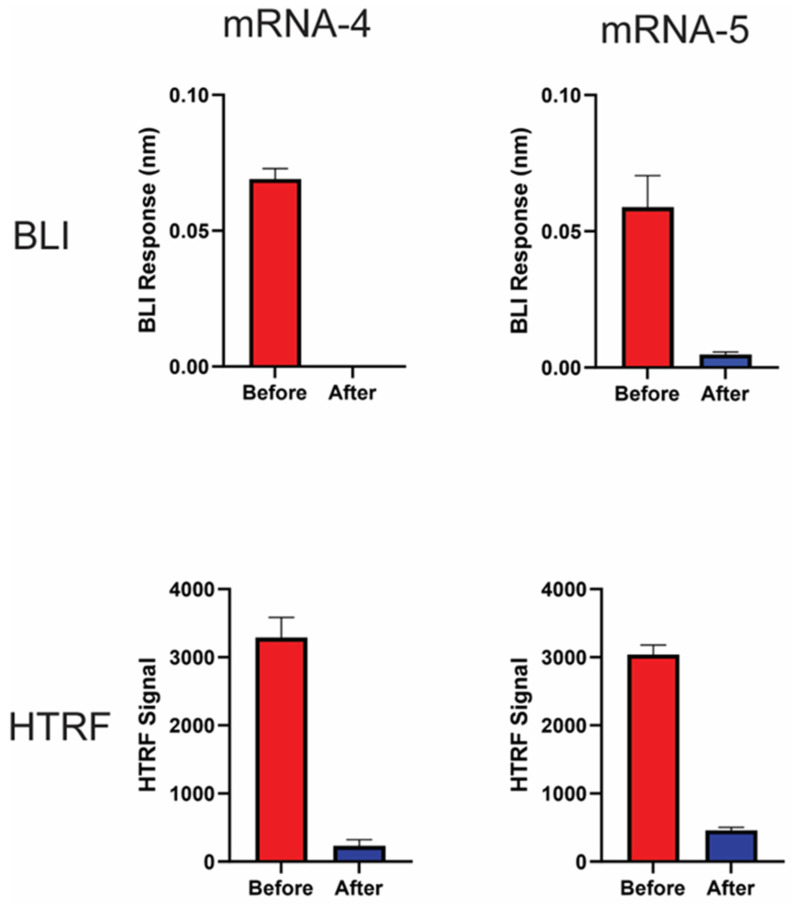
BLI dsRNA assay can monitor dsRNA reduction through purification process. BLI dsRNA detection assay utilized to monitor dsRNA level through purification process. Two IVT mRNA transcripts (mRNA-4 and mRNA-5) were purified using ion pair reverse-phase (IPRP) chromatography. Before: IVT mRNA before IPRP purification. After: IVT mRNA purified by IPRP. (**Top**) panel: BLI response of mRNA-4 and mRNA-5 tested at 20 µg/mL in triplicates (with blank subtracted). (**Bottom**) panel: HTRF signal of mRNA-4 and mRNA-5 tested at 10 µg/mL in triplicates (with blank subtracted).

Ion pair reverse-phase (IPRP) purification has been demonstrated as a reliable way of removing dsRNA impurities from IVT mRNA and reducing immunogenicity [17]. To evaluate whether the BLI dsRNA detection assay was able to monitor the dsRNA clearance after IPRP purification, two IVT mRNA samples (mRNA-4 and mRNA-5) were subjected to IPRP purification, and then the dsRNA levels of the samples before and after purification were assessed using both BLI dsRNA detection and HTRF assays. As expected, a nearly 100% reduction in dsRNA level was observed for both mRNA-4 and mRNA-5 following IPRP purification, as shown by BLI assay (Figure 7, Top). A highly similar trend was also observed when using the HTRF assay (Figure 7, Bottom), confirming that BLI dsRNA detection assay is a suitable in-process method to monitor the removal of dsRNA impurities during purification of IVT mRNA.

## 4. Discussion

In this study, a novel, rapid, and quantitative BLI dsRNA detection assay was developed to detect dsRNA impurities in IVT mRNA. As part of the characterization, the binding affinities of B2 for dsRNA with uridine modifications were systematically determined. Interestingly, the trend of binding affinity coincides with the degree of modification on the uridine (5 moU has the most significant modification while ψ has the least modification in terms of chemical changes). It is known that uridine modification can affect base-pairing interaction [31], which could potentially cause weakened binding to B2. These findings may help to provide further explanation, at least partially, as to why mRNA with modified uridine triggers lower innate immune response [32]. It is worth pointing out that this BLI method could be used to measure the binding affinity of B2 protein to both long and short dsRNA and, in this study, we only used 700 bp-dsRNA as an example. It will also be of great interest to apply this method for future studies to measure the binding affinity of the dsRNA binding domain (dsRBD) from PKR or other cellular dsRNA sensors towards dsRNA with or without modification.

It Is not just the B2–dsRNA interaction that can be impacted by the uridine modifications. Two commonly used antibodies, namely J2 and K1, which are the critical antibody reagents in various ELISA kits for dsRNA quantitation, also showed weaker binding towards dsRNA with modifications (Supplementary Table S3A,B). Although anti-dsRNA antibodies like J2 and K1 were isolated more than 30 years ago, this is the first study, to our knowledge, that measured the K_D_ values of J2 and K1 antibodies binding to dsRNA with or without modifications. For the J2 antibody, the impact of uridine modification on binding affinity followed the same trend as for B2 but with a more modest difference (5-fold vs. 15-fold for 5 moU, for example). It is worth noting that the K_D_ value determined for the J2 antibody using the BLI method is the overall affinity from the two Fab arms, not the affinity from an individual Fab arm, probably resulting in the less dramatic impact on the binding affinity values due to the avidity effect. In addition, the J2 antibody exhibited much stronger binding towards dsRNA when compared with the K1 antibody, which was consistent with earlier qualitative reports that the interaction between K1 and dsRNA was weaker [20,22,29]. The binding affinity of the K1 antibody for dsRNA with modification was also weaker compared with dsRNA without modification. In a recently published LFSA study [22], the author noticed the detection sensitivity for m1ψ dsRNA is much worse compared with U-containing dsRNA when using a combination of K1 (capture) and J5 (detection) antibodies. This observation could be partially attributed to the weaker affinity of the K1 antibody for m1ψ dsRNA, as demonstrated in our study. It will be interesting, in a follow-up study, to measure the binding affinity of the J5 antibody for dsRNA with and without modification to see whether this weaker affinity for modified dsRNA also applies to another anti-dsRNA antibody.

This difference in binding affinity could impact more than the detection sensitivity of the assay. For current commercially available dsRNA quantitation assays such as HTRF and ELISA, the dsRNA standards used for quantitation are premade and without uridine modification. Using this generic standard will cause under-reporting of dsRNA impurity levels for mRNA with uridine modification. As illustrated in Figure 5B,C, the HTRF assay exhibited a higher signal towards unmodified dsRNA when compared with dsRNA with uridine modifications. It is worth noting that HTRF does not the J2 or K1 antibodies. Instead, it uses a mouse antibody isolated in the 1980s [25]. This implies that the weaker binding affinity towards m1ψ- or 5 moU-modified dsRNA might be a general property of anti-dsRNA antibodies. For J2 ELISA, the right-shift of standard curves for m1ψ- or ψ-modified dsRNA indicated a much-reduced detection sensitivity and would potentially cause more than 10-fold under-reporting if using unmodified dsRNA standards to measure the levels of m1ψ- or ψ-modified dsRNA impurities.

To overcome the above-illustrated challenges from the impact of uridine modification on the dsRNA interaction while maintaining a simple-to-implement assay, avidity of binding was utilized to mitigate the effect of reduced binding affinity when designing the BLI assay for dsRNA detection. In our design, the biosensor surface immobilized with a saturating amount of B2 greatly reduced the impact of the weaker binding, resulting in similar detection sensitivities for dsRNA with or without uridine modification (Figure 5A and Supplementary Table S6). If accurate quantitation is the goal, given the difference in the high asymptote of the standard curves generated using dsRNA with different uridine modification (Figure 5A and Figure S4), it is still recommended to use dsRNA standard with the same uridine modification as used in the IVT process for producing mRNA. It was also interesting to observe a much-reduced dynamic range (signal between high asymptote and low asymptote) if B2 was replaced by the J2 or K1 antibody in the BLI dsRNA detection assay (Supplementary Figure S5), highlighting the importance of a dense capture surface for quantitation.

From our study, the BLI dsRNA detection assay also demonstrated the ability to detect and quantify dsRNA as short as 25 bp (U or m1ψ) (Figure 6 and Supplementary Figure S4), while the generally recognized length limit for J2 or K1 antibody-based detection is 40 bp. Indeed, a minimal signal was observed when testing 25 bp dsRNA standards on HTRF assay or J2 ELISA (Figure 6B,C). BLI dsRNA detection assay can also detect and quantify hairpin dsRNA (Figure 6A and Supplementary Figure S4), which contains a dsRNA loop formed by complementary regions before and after poly(A) tail, mimicking the loop-back dsRNA impurities from IVT process [3,14]. At the same time, both HTRF and J2 ELISA failed to detect such a loop-back dsRNA structure based on our testing (Figure 6B,C). The BLI dsRNA detection assay’s ability to detect shorter dsRNA as well as loop-back dsRNA might provide a more complete picture of dsRNA impurities in IVT-produced mRNA.

Lastly, we demonstrated that this BLI dsRNA detection assay can successfully monitor the dsRNA removal through a purification process. Compared with the ELISA or HTRF methods, which typically take 4–6 h or overnight to run, the rapid run time of the BLI dsRNA detection assay (35 min), BLI’s built-in automation, and the BLI dsRNA detection assay’s flexibility to use 96- or 384-well plates could make the BLI dsRNA detection assay an ideal at-line test to implement for monitoring dsRNA impurities between process steps. Given Octet^®^ instruments are 21CFR part 11-compliant, this assay could be qualified for release testing as well.

## 5. Conclusions

In this study, we developed a rapid, sensitive, and easy-to-implement dsRNA detection assay utilizing the BLI platform. This assay exhibited similar high sensitivity for dsRNA with various uridine modifications, contrary to the existing ELISA, HTRF, or LFSA methods. Besides detecting various modifications, dsRNAs as short as 25 bp and hairpin dsRNA can also be detected and quantified using this BLI assay, further demonstrating the potential wide application of this assay. Potential future work includes qualification of this method to prepare for QC and release testing. Overall, this BLI assay offers a more comprehensive detection for dsRNA of different modifications and structure features and would be an ideal assay to implement for IVT mRNA manufacturing.

## Figures and Tables

**Table 1:** Binding affinity of B2 for different 700 bp dsRNA standards.

dsRNA Binder	dsRNA	K_D_ (nM)	R_max_ (nm)
B2	700 bp-U	62 ± 4	0.545 ± 0.010
	700 bp-ψ	405 ± 20	0.604 ± 0.010
	700 bp-m1ψ	669 ± 26	0.605 ± 0.009
	700 bp-5 moU	948 ± 44	0.531 ± 0.011

K_D_ and R_max_ mean values and standard error of mean (SEM) were obtained from global steady-state analysis of three independent replicates. BLI signals from 290 s to 295 s were average to be used as binding response. Model equation for the steady-state analysis: *Response* = (*Rmax*×*Conc*)⁄((*KD* + *Conc*)). Analysis was performed using Graphpad Prism.

## Data Availability

The original contributions presented in the study are included in the article/Supplementary Materials.

## Supplementary Materials

The following supporting information can be downloaded at: https://www.mdpi.com/article/10.3390/pharmaceutics16091227/s1.

## References

[1] Pardi N., Hogan M.J., Porter F.W., Weissman D. (2018). MRNA Vaccines—A New Era in Vaccinology. Nat. Rev. Drug Discov..

[2] Sahin U., Karikó K., Türeci Ö. (2014). MRNA-Based Therapeutics—Developing a New Class of Drugs. Nat. Rev. Drug Discov..

[3] Mu X., Greenwald E., Ahmad S., Hur S. (2018). An Origin of the Immunogenicity of in Vitro Transcribed RNA. Nucleic Acids Res..

[4] Konarska M.M., Sharp P.A. (1989). Replication of RNA by the DNA-Dependent RNA Polymerase of Phage T7. Cell.

[5] Cazenave C., Uhlenbeck O.C. (1994). RNA Template-Directed RNA Synthesis by T7 RNA Polymerase. Proc. Natl. Acad. Sci. USA.

[6] Triana-Alonso F.J., Dabrowski M., Wadzack J., Nierhaus K.H. (1995). Self-Coded 3’-extension of Run-off Transcripts Produces Aberrant Products during in Vitro Transcription with T7 RNA Polymerase. J. Biol. Chem..

[7] Krupp G. (1989). Unusual Promoter-Independent Transcription Reactions with Bacteriophage RNA Polymerases. Nucleic Acids Res..

[8] Donovan J., Rath S., Kolet-Mandrikov D., Korennykh A. (2017). Rapid RNase L–Driven Arrest of Protein Synthesis in the DsRNA Response without Degradation of Translation Machinery. RNA.

[9] Husain B., Mukerji I., Cole J.L. (2012). Analysis of High-Affinity Binding of Protein Kinase R to Double-Stranded RNA. Biochemistry.

[10] Chung H., Calis J.J.A., Wu X., Sun T., Yu Y., Sarbanes S.L., Thi V.L.D., Shilvock A.R., Hoffmann H.-H., Rosenberg B.R. (2018). Human ADAR1 Prevents Endogenous RNA from Triggering Translational Shutdown. Cell.

[11] Loo Y.-M., Fornek J., Crochet N., Bajwa G., Perwitasari O., Martinez-Sobrido L., Akira S., Gill M.A., García-Sastre A., Katze M.G. (2008). Distinct RIG-I and MDA5 Signaling by RNA Viruses in Innate Immunity. J. Virol..

[12] Kato H., Takeuchi O., Sato S., Yoneyama M., Yamamoto M., Matsui K., Uematsu S., Jung A., Kawai T., Ishii K.J. (2006). Differential Roles of MDA5 and RIG-I Helicases in the Recognition of RNA Viruses. Nature.

[13] Karikó K., Buckstein M., Ni H., Weissman D. (2005). Suppression of RNA Recognition by Toll-like Receptors: The Impact of Nucleoside Modification and the Evolutionary Origin of RNA. Immunity.

[14] Dousis A., Ravichandran K., Hobert E.M., Moore M.J., Rabideau A.E. (2023). An Engineered T7 RNA Polymerase That Produces MRNA Free of Immunostimulatory Byproducts. Nat. Biotechnol..

[15] Wu M.Z., Asahara H., Tzertzinis G., Roy B. (2020). Synthesis of Low Immunogenicity RNA with High-Temperature in Vitro Transcription. RNA.

[16] Piao X., Yadav V., Wang E., Chang W., Tau L., Lindenmuth B.E., Wang S.X. (2022). Double-Stranded RNA Reduction by Chaotropic Agents during in Vitro Transcription of Messenger RNA. Mol. Ther.-Nucleic Acids.

[17] Karikó K., Muramatsu H., Ludwig J., Weissman D. (2011). Generating the Optimal MRNA for Therapy: HPLC Purification Eliminates Immune Activation and Improves Translation of Nucleoside-Modified, Protein-Encoding MRNA. Nucleic Acids Res..

[18] Romanovskaya A., Sarin L.P., Bamford D.H., Poranen M.M. (2013). High-Throughput Purification of Double-Stranded RNA Molecules Using Convective Interaction Media Monolithic Anion Exchange Columns. J. Chromatogr. A.

[19] Baiersdörfer M., Boros G., Muramatsu H., Mahiny A., Vlatkovic I., Sahin U., Karikó K. (2019). A Facile Method for the Removal of DsRNA Contaminant from In Vitro-Transcribed MRNA. Mol. Ther.-Nucleic Acids.

[20] Schonborn J., Oberstraβ J., Breyel E., Tittgen J., Schumacher J., Lukacs N. (1991). Monoclonal Antibodies to Double-Stranded RNA as Probes of RNA Structure in Crude Nucleic Acid Extracts. Nucleic Acids Res..

[21] Fujita M., Adachi K., Nagasawa M. (2019). Development of a Homogeneous Time-Resolved Fluorescence Assay for Detection of Viral Double-Stranded RNA. Anal. Biochem..

[22] Luo D., Wu Z., Wang D., Zhang J., Shao F., Wang S., Cestellos-Blanco S., Xu D., Cao Y. (2023). Lateral Flow Immunoassay for Rapid and Sensitive Detection of DsRNA Contaminants in in Vitro-Transcribed MRNA Products. Mol. Ther.-Nucleic Acids.

[23] Arney J.W., Weeks K.M. (2022). RNA–Ligand Interactions Quantified by Surface Plasmon Resonance with Reference Subtraction. Biochemistry.

[24] Lostao A., Lim K., Pallarés M.C., Ptak A., Marcuello C. (2023). Recent Advances in Sensing the Inter-Biomolecular Interactions at the Nanoscale—A Comprehensive Review of AFM-Based Force Spectroscopy. Int. J. Biol. Macromol..

[25] Kitagawa Y., Okuhara E. (1981). Anti-Poly(I)·poly(C) Antibody Bound to Cellulose and Its Use in the Specific Separation of Double-Stranded RNAs. Anal. Biochem..

[26] Li H., Li W.X., Ding S.W. (2002). Induction and Suppression of RNA Silencing by an Animal Virus. Science.

[27] Chao J.A., Lee J.H., Chapados B.R., Debler E.W., Schneemann A., Williamson J.R. (2005). Dual Modes of RNA-Silencing Suppression by Flock House Virus Protein B2. Nat. Struct. Mol. Biol..

[28] Lingel A., Simon B., Izaurralde E., Sattler M. (2005). The Structure of the Flock House Virus B2 Protein, a Viral Suppressor of RNA Interference, Shows a Novel Mode of Double-stranded RNA Recognition. EMBO Rep..

[29] Monsion B., Incarbone M., Hleibieh K., Poignavent V., Ghannam A., Dunoyer P., Daeffler L., Tilsner J., Ritzenthaler C. (2018). Efficient Detection of Long DsRNA in Vitro and in Vivo Using the DsRNA Binding Domain from FHV B2 Protein. Front. Plant Sci..

[30] Cheng X., Deng P., Cui H., Wang A. (2015). Visualizing Double-Stranded RNA Distribution and Dynamics in Living Cells by DsRNA Binding-Dependent Fluorescence Complementation. Virology.

[31] Chen Y.G., Hur S. (2022). Cellular Origins of DsRNA, Their Recognition and Consequences. Nat. Rev. Mol. Cell Biol..

[32] Anderson B.R., Muramatsu H., Nallagatla S.R., Bevilacqua P.C., Sansing L.H., Weissman D., Karikó K. (2010). Incorporation of Pseudouridine into MRNA Enhances Translation by Diminishing PKR Activation. Nucleic Acids Res..

