# Supplementary material for: Development of Biolayer Interferometry (BLI)-Based Double-Stranded RNA Detection Method with Application in mRNA-Based Therapeutics and Vaccines
Source: Pharmaceutics. 2024 Sep 19;16(9):1227. doi: 10.3390/pharmaceutics16091227 (PMC11435032; doi:10.3390/pharmaceutics16091227)
Supplement: Supplementary file 1 [file pharmaceutics-16-01227-s001.zip › pharmaceutics-3163511-supplementary.pdf]

Supplemental Tables and Figures

Supplemental Table S1. List of dsRNA standards used in this study.

dsRNA	Description	Estimated dsRNA length^	M.W. (kDa)
25bp-U	Short dsRNA*, uridine	25 bp	16.0
25bp-m1ψ	Short dsRNA*, m1ψ	25 bp	16.1
700bp-U	dsRNA based on GLuc mRNA, uridine	557 bp	367.6
700bp-m1ψ	dsRNA based on GLuc mRNA, m1ψ	557 bp	371.4
700bp-ψ	dsRNA based on GLuc mRNA, ψ	557 bp	367.6
700bp-5moU	dsRNA based on GLuc mRNA, 5moU	557 bp	378.2
1800bp-U	dsRNA based on FLuc mRNA, uridine	1652 bp	1090.3
1800bp-m1ψ	dsRNA based on FLuc mRNA, m1ψ	1652 bp	1098.7
Hairpin-U	642-nt mRNA with poly(A) tail 70-bp complementary strand before and after poly(A), uridine	70 bp	291.7
Hairpin-m1ψ	642-nt mRNA with poly(A) tail 70-bp complementary strand before and after poly(A), m1ψ	70 bp	293.4

* 25bp dsRNA sequence as follows: (sense) 5' CACGUACCGUUCUAGCGGGCUCUCG 3'; (anti-sense) 3' GUGCAUGGCAAGAUCGCCCGAGAGC 5'

^ Estimated based on sequence complementarity.

Supplemental Table S2. dsRNA purity analysis by capillary electrophoresis.

**dsRNA samples**	**Purity[#] (CPA%)**
25bp-U	89.4
25bp-m1ψ	85.4
700bp-U	96.5
700bp-m1ψ	98.1
700bp-ψ	99.2
700bp-5moU	91.8
1800bp-U	96.6
1800bp-m1ψ	94.5
Hairpin-U	87.3
Hairpin-m1ψ	84.6

The corrected peak area % (CPA %) values are an average of duplicate runs.

Supplemental Table S3A. J2 antibody binding affinity to dsRNA with different uridine modification.

dsRNA binder	dsRNA	K_D (nM)	k_{on} (1/Ms)	k_{off} (1/s)
J2	700bp-U	27 ± 3	$(2.11 \pm 0.21)\ E^{5}$	$(5.65 \pm 0.09)\ E^{-3}$
	700bp-ψ	32 ± 5	$(1.89 \pm 0.50)\ E^{5}$	$(6.03 \pm 1.05)\ E^{-3}$
	700bp-m1ψ	56 ± 1	$(1.22 \pm 0.02)\ E^{5}$	$(6.80 \pm 0.18)\ E^{-3}$
	700bp-5moU	131 ± 5	$(8.68 \pm 0.51)\ E^{4}$	$(1.14 \pm 0.08)\ E^{-2}$

K_D, k_{on} and k_{off} mean values and standard error of mean (SEM) were obtained from three independent replicates.

Supplemental Table S3B. K1 antibody binding affinity to dsRNA with different uridine modification.

dsRNA binder	dsRNA	K_D (nM)
K1	700bp-U	325 ± 33
	700bp-ψ	µM range
	700bp-m1ψ	
	700bp-5moU	

K_D, k_{on} and k_{off} mean values and standard error of mean (SEM) were obtained from three independent replicates.

Model fitting 1: 1 binding model. Analysis was performed using Graphpad Prism.

Supplemental Table S4. Welch's T-test analysis of BLI dsRNA detection assay interference testing.

Group A	Group B	P value	Significant different (P < 0.05)
700bp-U	100X ssRNA	0.3465	No
	200X ssRNA	0.6897	No
	100X dsDNA	0.1065	No
	200X dsDNA	0.1501	No
700bp-m1ψ	100X ssRNA	0.5802	No
	200X ssRNA	0.5593	No
	100X dsDNA	0.3612	No
	200X dsDNA	0.0113	Yes

Supplemental Table S5. Representative fitting parameters for dsRNA standards for BLI dsRNA detection assay.

dsRNA standards	Bottom (nm)	Top (nm)	Hillslope	EC50 (ng/mL)
700bp-U	-0.006067	0.7703	1.023	204.6
700bp-m1ψ	0.002437	0.6377	1.110	191.7
700bp-5moU	-0.009762	0.6802	0.965	211.0
700bp-ψ	-0.001121	0.6843	0.972	204.8

Supplemental Table S6. Limits of detection and limit of quantitation for different dsRNA standards determined for BLI dsRNA detection method.

dsRNA binder	dsRNA	Limit of detection[1] (ng/mL)	Limit of quantitation[2] (ng/mL)
B2	700bp-U	14.0	59.5
	700bp-ψ	13.7	64.6
	700bp-m1ψ	27.9	89.7
	700bp-5moU	15.9	70.0
	25bp-U	9.3	41.2
	25bp-m1ψ	21.5	154.6
	1800bp-U	23.8	74.0
	1800bp-m1ψ	35.2	94.0
	Hairpin-U	17.4	58.6
	Hairpin-m1ψ	16.1	84.0

[1] The LOD is defined as the lowest dsRNA concentration giving a signal greater than the nonspecific binding (mean of six or twelve measurements of BLI assay buffer) + 3x standard deviations (six or twelve measurements).

[2] The LOQ is defined as the lowest dsRNA concentration giving a signal greater than the nonspecific binding (mean of six or twelve measurements of BLI assay buffer) + 10x standard deviations (six or twelve measurements).

Supplemental Figure S1. Illustration of BLI method for dsRNA binding affinity measurement.

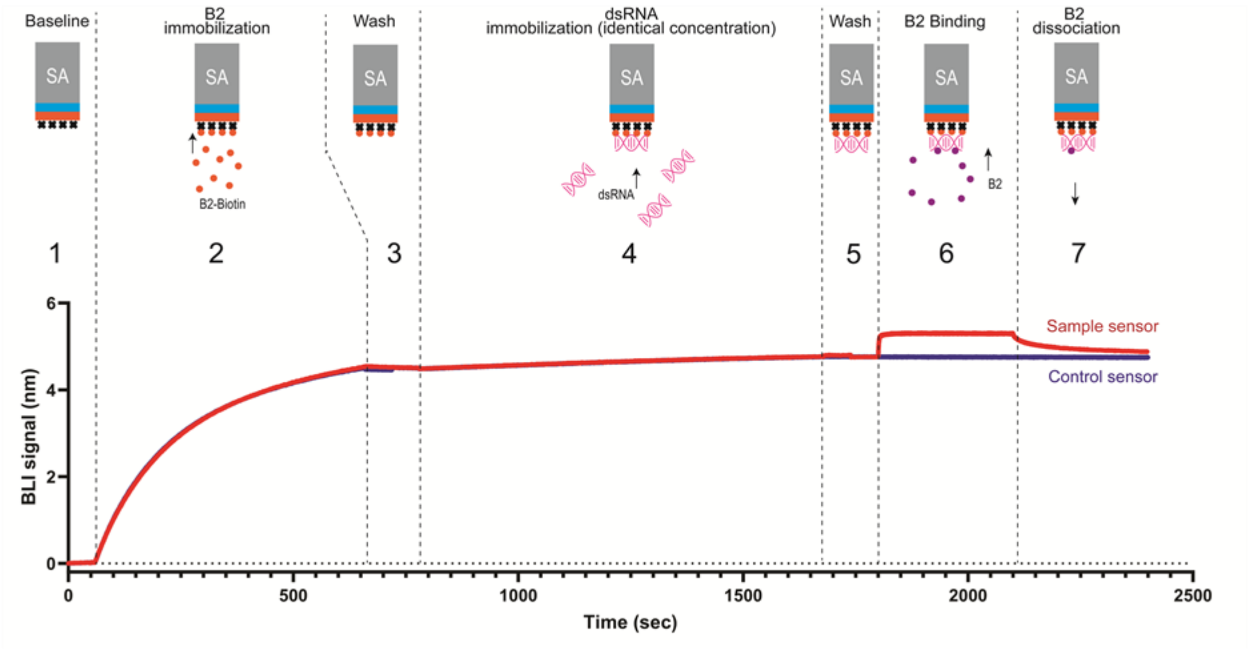

BLI method for measuring binding affinity between B2 protein and different dsRNA with representative sensorgrams. Each step is labeled with corresponding sensogram section. Step 1) Establish baseline in assay buffer; 2) Immobilize B2-Biotin on SA sensors; 3) wash to remove excess un-bound B2-Biotin; 4) Capture dsRNA on B2 surface; 5) wash to remove un-bound dsRNA; 6) serial diluted B2 binding to dsRNA surface; 8) B2 dissociation from dsRNA surface.

Supplemental Figure S2. Representative BLI dsRNA detection assay sensorgrams and standard curve.

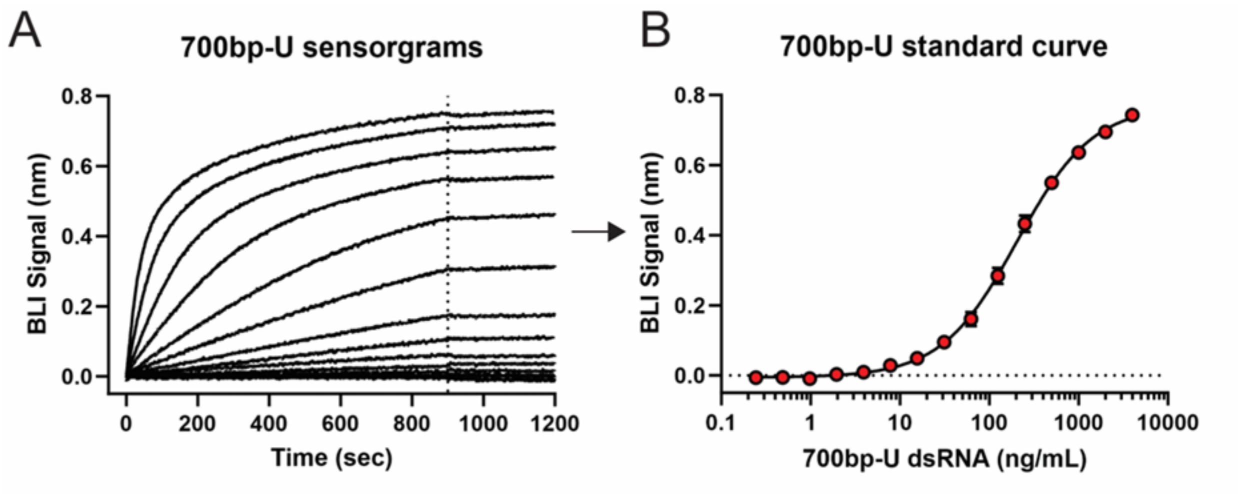

Representative 700bp-U dsRNA standard curve. A) sensorgrams of 700bp-U measured by BLI dsRNA detection assay; B) 700bp-U standard curve was generated by plotting BLI response at a given time frame against dsRNA concentration. The standard curve was fitted using a 4PL model.

Supplemental Figure S3. 700bp-m1ψ standard curve in the presence of excess ssRNA.

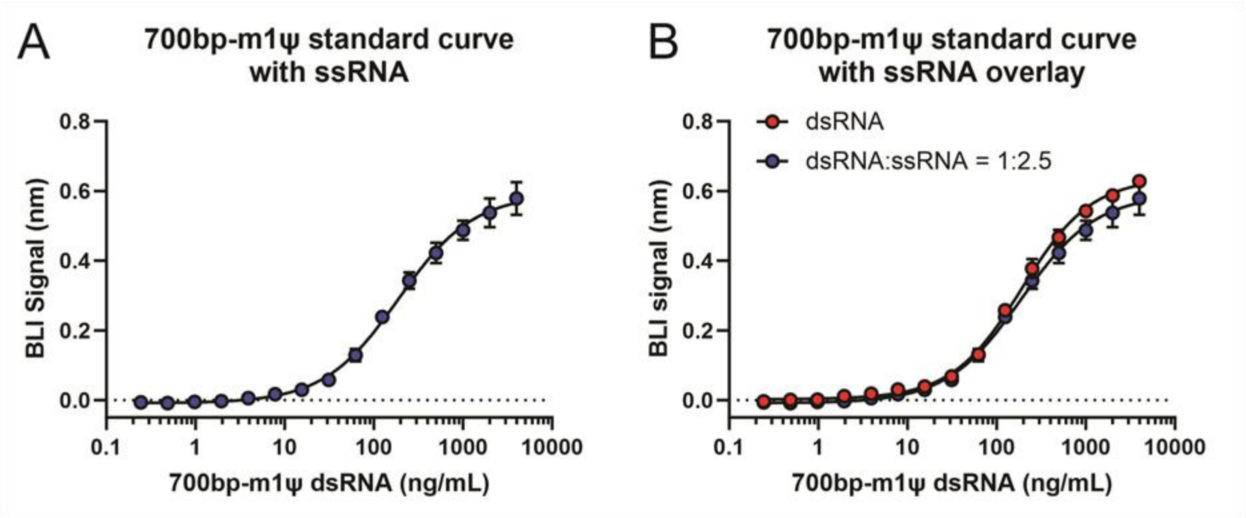

Comparison of 700bp-m1ψ standard curve with and without excessive amount of ssRNA. A) 700bp-m1ψ standard curve with ssRNA at a ratio of dsRNA: ssRNA = 1: 2.5. B) Overlay of 700bp-m1ψ standard curve with and without ssRNA. All measurements were performed in triplicate.

Supplemental Figure S4. dsRNA standard curve of different length on BLI method.

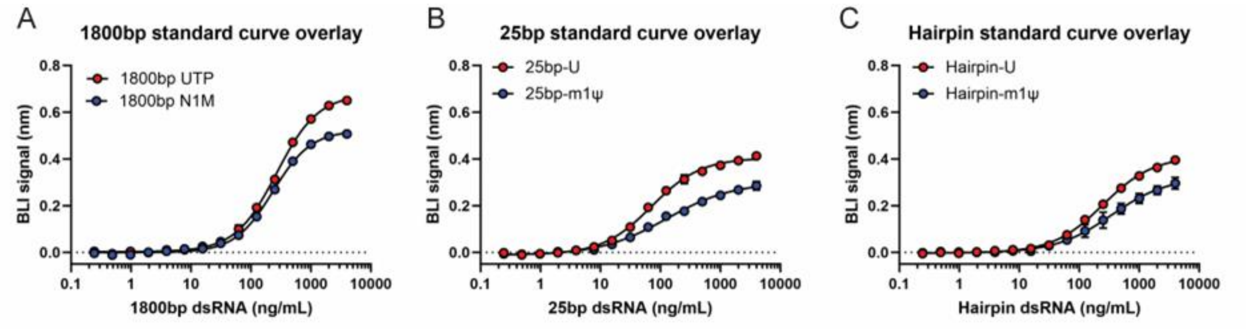

Comparison of dsRNA standard curve of different length. A) overlay of 1800bp (U or m1ψ) standard curves. B) overlay of 25bp (U or m1ψ) standard curves. C) overlay of hairpin (U or m1ψ) standard curves.

Supplemental Figure S5. Replace B2 in the BLI dsRNA detection assay with anti-dsRNA antibodies reduced assay dynamic range.

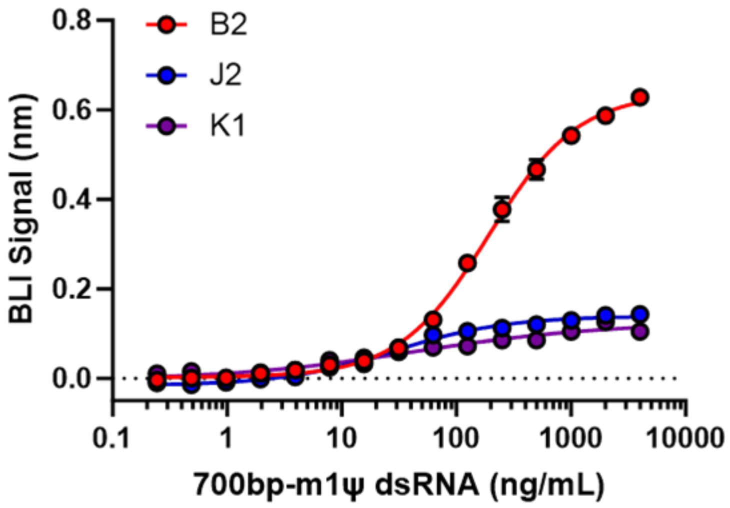

Replace B2 in BLI dsRNA assay with either J2 or K1 antibody. Overlay of standard curve (700bp-m1ψ) showed there is significant reduced dynamic range for the BLI dsRNA assay. All measurements were performed in triplicate.
